# Latent profiles of pre-competition psychological states in young Chinese judo athletes: associations with depression and mental toughness

**DOI:** 10.3389/fpubh.2026.1921861

**Published:** 2026-08-13

**Authors:** Yongsheng Zhang, Zhining Niu, Zongyu Liu, Wenjia Chen, Ruihan Chen, Haitao Niu

**Affiliations:** 1 School of Art and Design, Weifang University of Science and Technology, Shouguang, Shandong, China; 2 School of Physical Education and Health, Guangxi Minzu University, Nanning, Guangxi, China; 3 Department of Sports Science, College of Education, Zhejiang University, Hangzhou, Zhejiang, China; 4 School of Physical Education, China University of Mining and Technology, Xuzhou, China; 5 School of Physical Education, Shandong University, Jinan, China

**Keywords:** competitive state anxiety, judo athletes, latent profile analysis, mental toughness, perceived social support, person-centered

## Abstract

**Background:**

Young judo athletes show highly heterogeneous pre-competition psychological states, yet prior variable-centered research has assumed a uniform linear “support–anxiety” relationship that masks individual differences. Using latent profile analysis, this study aimed to identify subgroups jointly defined by perceived social support and competitive state anxiety, examine unadjusted between-profile differences in depression and mental toughness, and investigate covariate-adjusted correlates of profile membership.

**Methods:**

A cross-sectional design was employed. A total of 222 young Chinese judo athletes were assessed using the Multidimensional Scale of Perceived Social Support (MSPSS), the Competitive State Anxiety Inventory-2 (CSAI-2), the Center for Epidemiologic Studies Depression Scale (CES-D), and the Sports Mental Toughness Questionnaire (SMTQ). Standardized perceived social support and competitive state anxiety served as the indicator variables for latent profile analysis (LPA); the retained profile solution was selected by jointly considering the AIC, BIC, SABIC, entropy, and class proportions. Binary logistic regression was used to examine the covariate-adjusted associations of mental toughness and habitual stress with profile membership while controlling for age, sex, and history of acute sports injury. Independent-samples *t*-tests were used to examine unadjusted between-profile differences in depression and mental toughness.

**Results:**

A two-profile solution was retained after jointly considering information criteria, profile size, classification accuracy, parsimony, and substantive interpretability (entropy = 0.679). The Resource-Deprived profile comprised 36.5% of the sample, whereas the Supported–Vigilant profile comprised 63.5%. The Supported–Vigilant profile reported lower depression (*p* < 0.001) and higher mental toughness (*p* = 0.035). Mental toughness was associated with higher adjusted odds of Supported–Vigilant profile membership (OR = 1.34, 95% CI [1.01, 1.80], *p* = 0.047).

**Conclusion:**

The retained profiles were distinguished primarily by jointly lower versus jointly higher levels of perceived social support and composite competitive state anxiety. In the Supported–Vigilant profile, higher composite competitive state anxiety coexisted with lower depression and higher mental toughness; however, the present findings do not establish that anxiety was facilitative or that social support buffered its effects. These findings suggest that pre-competition psychological work should assess anxiety intensity together with perceived support and broader psychological adjustment, rather than uniformly pursuing anxiety reduction, while giving particular attention to resource-deprived athletes.

## 1 Introduction

Competitive sport, particularly highly combative, weight-classified fighting disciplines such as judo, poses sustained physical and psychological challenges to athletes of all ages, including adolescents (1). Systematic reviews have noted that participation in combat sports is fairly consistently associated with benefits in certain cognitive functions (especially perception and inhibitory control), but evidence for its associations with mental illness and self-related outcomes remains relatively limited (2). At the same time, the pressures of training, weight cutting, injury, and competitive evaluation place this population at non-negligible risk for mental health problems (1). During adolescence, depression, stress, and anxiety are already common psychological difficulties (3), and the pressures specific to competitive environments make young athletes especially vulnerable to such problems; if not adequately addressed, these symptoms may persist into adulthood and evolve into more serious psychiatric disorders (4). Characterizing the pre-competition psychological states of young judo athletes and identifying populations in need of priority intervention therefore carries considerable practical significance.

Within combat sports, judo provides a particularly informative setting for examining pre-competition psychological states. Competitions are organized by weight category, and rapid weight loss before official weigh-ins is common among judo athletes; such practices may adversely affect mood, fatigue, and psychological well-being in the period surrounding competition (5, 6). Judo is also a direct one-on-one grappling sport characterized by intermittent high-intensity exchanges, continuous adaptation to an opponent, and the possibility that a single ippon can immediately end a contest. These features impose substantial demands on vigilance, pacing, and rapid technical–tactical decision making (7). In China, competitive results are additionally linked to a formal athlete technical-grade system, making competitive classification a salient component of athletes’ sporting development. Together, weight-management and weigh-in requirements, opponent-dependent tactical uncertainty, and formal competitive evaluation create a distinctive pre-competition context. These characteristics support examining young judo athletes specifically rather than assuming that findings from broader athlete samples apply uniformly to them.

Perceived social support is widely regarded as a core protective resource for athletes’ mental health. A systematic review and meta-analysis comprising 40 studies and 14,462 athletes reported that overall social support was positively associated with athlete well-being (*r* = 0.31) and negatively associated with anxiety (*r* = −0.22), depression (*r* = −0.27), and stress (*r* = −0.25), with support from family and friends providing a particularly pronounced buffering effect against depressive symptoms (8). At the mechanistic level, social support is thought to attenuate the negative psychological impact of stress by promoting emotion regulation, fostering adaptive coping strategies, and lowering subjective appraisals of the severity of stressful situations (8). In sport contexts, athletes who perceive strong social support are more inclined to appraise competition as a “challenge” rather than a “threat,” so social support both nurtures mental toughness and acts as a buffer against high-pressure situations (9, 10). A study of adolescent athletes further showed that, after controlling for age and competitive level, social support positively predicted sport engagement, an effect mediated by self-efficacy and mental toughness (11).

Mental toughness refers to an athlete’s capacity to manage stress and the associated anxiety in high-pressure competitive situations, and is regarded by athletes, coaches, and sport psychologists alike as a key psychological characteristic underlying competitive success (11, 12). Among elite athletes, resilience-related qualities can distinguish “high-resilience” and “moderate-resilience” profiles, with members of the high-resilience profile showing healthier health-related behaviors, greater well-being, higher perceived social support, and higher self-rated performance (13). This suggests that psychological resources such as social support and toughness may not be uniformly distributed across athlete populations but instead cluster in a configurational manner within distinct subgroups.

In contrast to “resource” variables such as social support and mental toughness, competitive state anxiety has traditionally been viewed as a negative factor that impairs performance (14). However, developments in sport psychology have shown that anxiety intensity and directional interpretation should be distinguished: similar levels of anxiety may be experienced as either facilitative or debilitative depending on how athletes interpret their symptoms (15, 16). Empirical findings support this distinction. Elite and non-elite competitive swimmers reported comparable levels of cognitive and somatic anxiety intensity, whereas elite swimmers were more likely to interpret these symptoms as facilitative and also reported greater self-confidence (17). Interview research with elite and subelite swimmers further suggested that perceived control over anxiety symptoms, rather than the presence of the symptoms alone, was central to whether anxiety was interpreted as facilitative or debilitative (18). Evidence from combat sports similarly indicates that self-confidence and the regulation of emotional arousal are associated with self-rated performance in a model involving challenge appraisal (19). Together, these findings suggest that elevated anxiety intensity does not necessarily imply poorer psychological functioning. Importantly, however, the present study assessed anxiety intensity rather than anxiety direction, perceived control, or challenge–threat appraisal. It therefore examined whether relatively elevated anxiety could coexist with stronger psychological resources and more favorable external correlates, rather than whether anxiety itself was experienced as facilitative. Such heterogeneity may be difficult to detect using variable-centered analyses alone, because athletes with relatively elevated anxiety and more favorable functioning may be obscured within average associations.

Person-centered latent profile analysis (LPA) offers a methodological path out of this impasse. Rather than asking “how does variable A generally affect variable B,” LPA identifies subgroups of individuals with distinct configurations across multiple indicators and estimates each individual’s probability of membership in each profile, thereby overcoming limitations of traditional cluster analysis (20). In research on athlete emotions, prior work based on pre-competition emotional intensity and direction has identified profiles such as “emotionally balanced,” “facilitative arousal,” and “low arousal,” with the facilitative-arousal profile reporting stronger challenge appraisals, greater perceived control, and more adaptive coping strategies (21). Such findings indicate that the meaningful heterogeneity in athletes’ pre-competition psychological states often becomes apparent only when resource variables and arousal variables are jointly considered through a configurational lens.

To clarify the psychological meaning of the identified profiles, depression and mental toughness were considered complementary external correlates of psychological adjustment. Depression was treated as an indicator of adverse psychological functioning, whereas mental toughness represented an adaptive psychological resource for coping with competitive demands (12). Because neither variable was used to define the latent profiles, differences in depression and mental toughness were examined to help clarify the substantive meaning of the configurations identified on the basis of perceived social support and competitive state anxiety (20). This approach allowed us to examine whether higher competitive anxiety was necessarily associated with poorer adjustment or whether it could coexist with lower depressive symptoms and greater mental toughness when accompanied by stronger perceived support (15, 16, 19, 21). Both variables were interpreted as correlates of profile membership.

In sum, the present study had three aims: first, to use LPA to identify the pre-competition psychological profiles of young Chinese judo athletes jointly defined by perceived social support and competitive state anxiety; second, to examine unadjusted differences between the identified profiles in depression and mental toughness as complementary external correlates of psychological adjustment; and third, to examine the covariate-adjusted associations of mental toughness, habitual stress, and demographic and athletic characteristics with profile membership. Based on the reviewed literature, we formulated two hypotheses. First, we hypothesized that more than one latent profile would emerge from the joint distribution of perceived social support and competitive state anxiety (H1). Second, if a profile combining relatively elevated competitive anxiety with stronger perceived social support emerged, we hypothesized that it would show lower depressive symptoms and higher mental toughness than a profile characterized by weaker perceived support (H2). Because the available evidence was insufficient to specify directional expectations for habitual stress or demographic and athletic characteristics, their covariate-adjusted associations with profile membership were examined exploratorily. Given the cross-sectional design, all associations were interpreted as correlational rather than causal.

## 2 Methods

### 2.1 Study design and participants

This study employed a cross-sectional online questionnaire design. Using a team-based, multisite recruitment strategy, young judo athletes were recruited through judo teams and organized training programs in 17 provincial-level regions across China between July and August 2024. Coaches or designated team contacts distributed the survey link to athletes in the participating teams. The participating regions were Beijing, Shanghai, Tianjin, Chongqing, Shandong, Henan, Guangdong, Sichuan, Inner Mongolia, Shanxi, Shaanxi, Liaoning, Zhejiang, Jiangsu, Guangxi, Hubei, and Hunan.

Eligibility criteria were (a) current membership in a participating judo team or organized training program, (b) age no older than 25 years, and (c) voluntary participation with valid informed consent. A total of 240 athletes were invited to participate, of whom 222 submitted the questionnaire, yielding a response rate of 92.5%. Submitted questionnaires were screened for incomplete responses, careless or patterned response sets, and completion times below 300 s. The online survey platform automatically recorded the elapsed time from questionnaire entry to final submission for each response; this system-generated duration was used to identify submissions completed in less than 300 s. All 222 submitted questionnaires satisfied the eligibility and data-quality criteria and were retained in the final analytical sample. The study was approved by the Ethics Committee of Shandong University (Approval No. ECSBMSSDU2021-1-114) and was conducted in accordance with the Declaration of Helsinki and applicable institutional requirements. Adult participants provided written informed consent. For participants younger than 18 years, written informed consent was obtained from a parent or legal guardian, and assent was obtained from the participant.

The final analytical sample comprised 222 young judo athletes aged 14–25 years (M = 18.97, SD = 2.68). The sample comprised 116 males (52.3%) and 106 females (47.7%), a relatively balanced sex ratio. Non-only-children constituted the majority (167, 75.2%), with only-children numbering 55 (24.8%). Monthly household income was predominantly below ¥10,000 (150, 67.6%), with 59 (26.6%) at ¥10,000–20,000 and only 13 (5.9%) above ¥20,000. With respect to judo grade, first-class athletes were the most numerous (93, 41.9%), followed by ungraded athletes (54, 24.3%) and national-level masters (45, 20.3%), with second-class athletes (27, 12.2%) and international-level masters (3, 1.4%). Regarding injury, 81 athletes (36.5%) reported an acute sports injury within the past year and 88 (39.6%) reported a chronic sports injury. Full demographic and athletic background characteristics are presented in Table 1.

**Table 1:** Demographic and athletic background characteristics of the sample (*N* = 222).

Characteristic	Category	*n*	%
Sex	Male / Female	116/106	52.3/47.7
Only child	Yes / No	55/167	24.8/75.2
Monthly household income	<¥10 k / ¥10–20 k / >¥20 k	150/59 / 13	67.6/26.6/5.9
Judo grade	Ungraded / Second-class / First-class / National master / International master	54/27 / 93/45 / 3	24.3/12.2/41.9/20.3/1.4
Acute injury (past year)	Yes / No	81/141	36.5/63.5
Chronic injury	Yes / No	88/134	39.6/60.4

### 2.2 Measures

#### 2.2.1 Perceived social support

The Multidimensional Scale of Perceived Social Support (MSPSS) (22) was used to measure individuals’ subjective perceptions of the social support they receive. The scale comprises 12 items spanning three dimensions, support from family, friends, and significant others, with 4 items each. All items are scored on a 7-point Likert scale (1 = *very strongly disagree*, 7 = *very strongly agree*); dimension scores and a total score can be computed, with higher total scores indicating stronger perceived social support. The scale has demonstrated good reliability and validity in cross-cultural research and has been validated in Chinese samples (23). In the present study, Cronbach’s *α* = 0.957 and McDonald’s *ω* = 0.958, indicating excellent internal consistency.

#### 2.2.2 Competitive state anxiety

The Competitive State Anxiety Inventory-2 (CSAI-2) (24) was used to assess athletes’ pre-competition state anxiety. The inventory comprises 27 items across three dimensions, cognitive state anxiety, somatic state anxiety, and state self-confidence, with nine items each, respectively measuring cognitive components such as worry about failure before competition, physiological arousal components such as increased heart rate and muscle tension, and confidence in one’s own competitive performance. Items are rated on a 4-point intensity scale reflecting the strength of the corresponding feeling. In the present study, the composite total competitive state anxiety score derived from the three dimensions served as the classification indicator. The inventory has been validated in Chinese samples (25). In the present study, Cronbach’s *α* = 0.880 and McDonald’s *ω* = 0.892, indicating good internal consistency.

#### 2.2.3 Depression

The Center for Epidemiologic Studies Depression Scale (CES-D) (26) was used to assess the severity of depressive symptoms. The scale comprises 20 items asking respondents to report the frequency of various depression-related feelings and behaviors over the past week, scored on a 4-point scale (from “rarely or none of the time” to “most or all of the time”), with higher scores indicating more severe depressive symptoms. The scale is widely used for depression screening in non-clinical populations and has been validated in Chinese samples (27). In the present study, Cronbach’s *α* = 0.830 and McDonald’s *ω* = 0.872, indicating acceptable internal consistency.

#### 2.2.4 Mental toughness

The Sports Mental Toughness Questionnaire (SMTQ) (28) was used to assess confidence, constancy, and control that athletes display when facing competitive pressure, adversity, and setbacks. The questionnaire comprises 14 items scored on a 5-point scale, with higher scores indicating higher mental toughness and more abundant psychological resources for coping with stressful situations. The Chinese adaptation of the questionnaire has been used in Chinese athlete samples (29). In the present study, Cronbach’s *α* = 0.808 and McDonald’s *ω* = 0.822, indicating acceptable internal consistency.

### 2.3 Statistical analysis

Analyses were conducted in R version 4.4.1. Latent profile analysis was performed using the tidyLPA package, which calls mclust internally. Binary logistic regression, independent-samples *t*-tests, and chi-square tests were conducted using the stats package, and the broom package was used to extract regression estimates and confidence intervals. Standardized (*z*-score) perceived social support and competitive state anxiety served as the indicator variables. Standardization was intended to eliminate the scaling inconsistency arising from differences in the number of items and response metrics across the two scales, ensuring that the indicators carried balanced weight in the classification decision. A Gaussian mixture model with equal variances and zero covariances was applied, with the number of profiles increased incrementally starting from a single profile. Model selection jointly considered the Akaike Information Criterion (AIC), Bayesian Information Criterion (BIC), and sample-size-adjusted BIC (SABIC), with lower values indicating better relative fit; entropy, with values closer to 1 indicating clearer profile separation; profile size and stability; and substantive interpretability and parsimony. Entropy was treated as a diagnostic of classification quality rather than as a stand-alone selection criterion. Solutions containing a profile representing less than 5% of the sample were considered potentially unstable.

For the auxiliary analyses, participants were assigned to the profile with the highest posterior probability. Depression and mental toughness were treated as external correlates of profile membership. Independent-samples *t*-tests were used to examine unadjusted between-profile differences in depression and mental toughness. A binary logistic regression model, with profile membership as the dependent variable, was used to examine the covariate-adjusted association between mental toughness and profile membership while controlling for habitual stress (assessed using a single item, “How much stress do you generally feel?,” with response options of high, moderate, and low), age, sex, and history of acute sports injury. Mental toughness, habitual stress, and age were z-standardized to facilitate comparison of the odds ratios (ORs). Given the cross-sectional design, the regression coefficients were interpreted as adjusted associations rather than evidence of temporal prediction or causation. Chi-square tests were used to compare the distribution of profile membership across sex, only-child status, monthly household income, judo grade, and histories of acute and chronic sports injuries. Only-child status was included as an exploratory, culturally relevant background variable because China’s historical family-planning context has made sibling status a salient aspect of family structure, potentially corresponding to differences in parental attention, resource allocation, and perceived support; no directional hypothesis was specified.

## 3 Results

### 3.1 Descriptive statistics and correlations

Descriptive statistics and the correlation matrix for the main variables are presented in Table 2. Athletes’ perceived social support (*M* = 5.44, *SD* = 1.16) and competitive state anxiety (*M* = 2.54, *SD* = 0.36) were, overall, at moderately high levels; depression scores were low (*M* = 1.68, *SD* = 0.40), and mental toughness was at a moderate level (*M* = 2.97, *SD* = 0.55). Correlation analysis showed that perceived social support was moderately positively correlated with competitive state anxiety (*r* = 0.56, *p* < 0.001), suggesting that in this sample, athletes with more abundant social support also reported higher composite competitive state anxiety scores. Perceived social support was moderately negatively correlated with depression (*r* = −0.39, *p* < 0.001) and weakly positively correlated with mental toughness (*r* = 0.15, *p* < 0.05); competitive state anxiety was positively correlated with mental toughness (*r* = 0.29, *p* < 0.001), but its correlation with depression was not significant (*r* = 0.06, *p* > 0.05). Mental toughness and depression were likewise not significantly correlated (*r* = 0.11, *p* > 0.05).

**Table 2:** Descriptive statistics and correlation matrix for the main variables.

Variable	*M*	*SD*	1	2	3	4
1 Perceived social support	5.44	1.16	1			
2 Competitive state anxiety	2.54	0.36	0.56***	1		
3 Depression	1.68	0.40	−0.39***	0.06	1	
4 Mental toughness	2.97	0.55	0.15*	0.29***	0.11	1

*p < 0.05; ***p < 0.001.

### 3.2 Determination of the latent profiles

**Figure 1:**
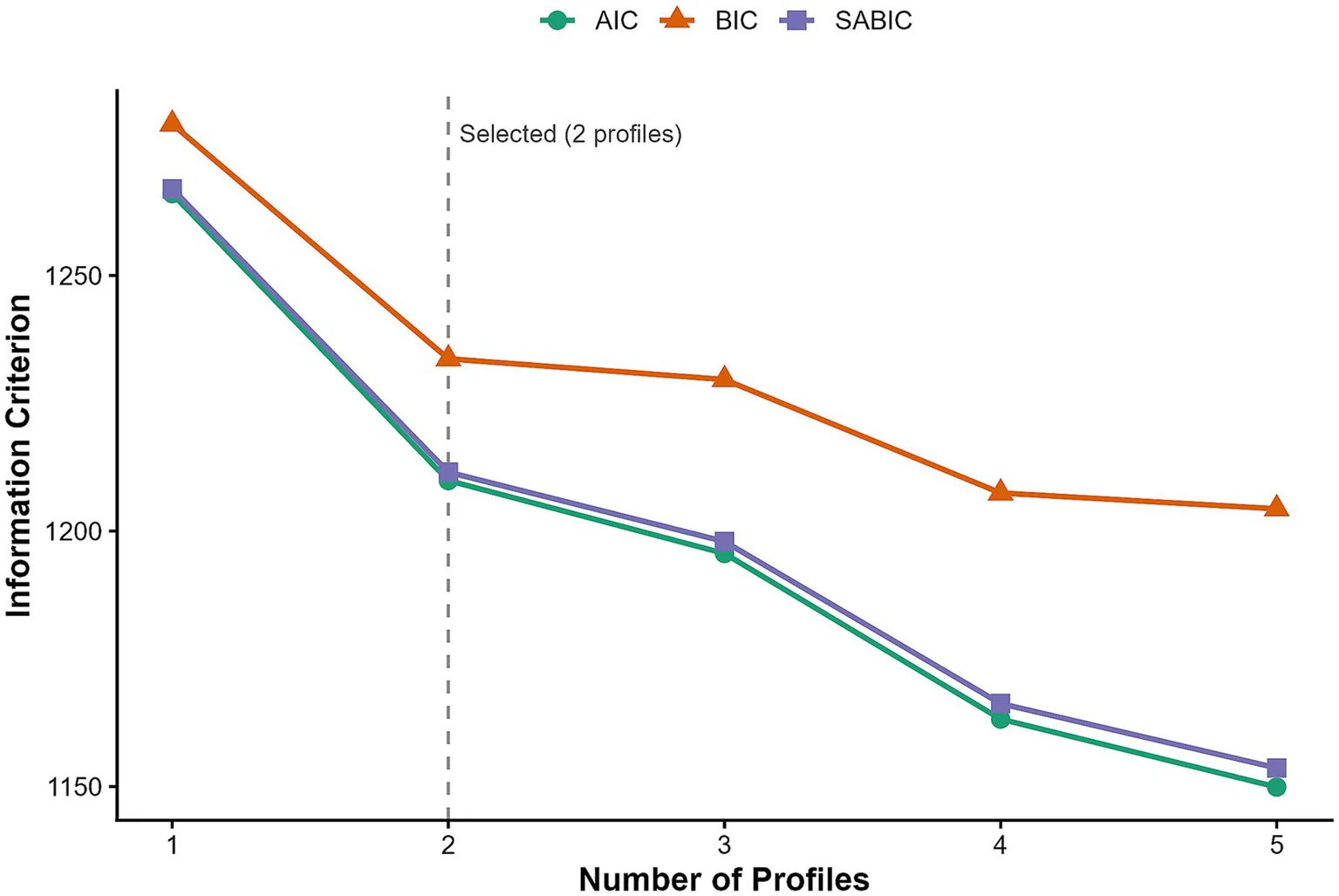
Information criteria (AIC, BIC, SABIC) across one- to five-profile solutions.

Models with one to five profiles were enumerated; fit indices are presented in Table 3 and Figure 1. The information criteria generally decreased as the number of profiles increased. However, the three- to five-profile solutions each produced an extremely small profile containing approximately 1.4% of the sample (approximately three athletes), raising concerns about profile stability and substantive interpretability. In contrast, the two-profile solution produced two adequately sized profiles, showed moderate classification accuracy (entropy = 0.679), and yielded a parsimonious and substantively interpretable configuration. Considering the information criteria, profile proportions, classification accuracy, parsimony, and theoretical interpretability together, the two-profile solution was retained. The smaller profile comprised 36.5% of the sample, well above the prespecified 5% threshold.

**Table 3:** Fit indices for the enumerated latent profile models (indicators = perceived social support + competitive state anxiety).

No. of profiles	LogLik	AIC	BIC	SABIC	Entropy	Smallest proportion	Largest proportion
1	−629.01	1266.01	1279.62	1266.95	1.000	1.000	1.000
2	−597.94	1209.88	1233.70	1211.52	0.679	**0.365**	0.635
3	−587.81	1195.61	1229.64	1197.95	0.792	0.014	0.667
4	−568.61	1163.21	1207.45	1166.25	0.869	0.014	0.577
5	−558.95	1149.91	1204.35	1153.65	0.847	0.014	0.392

Bold indicates the proportion of the smaller profile in the retained two-profile solution. The smallest profiles in the three- to five-profile solutions each represented approximately 1.4% of the sample.

### 3.3 Profile characteristics and naming

**Figure 2:**
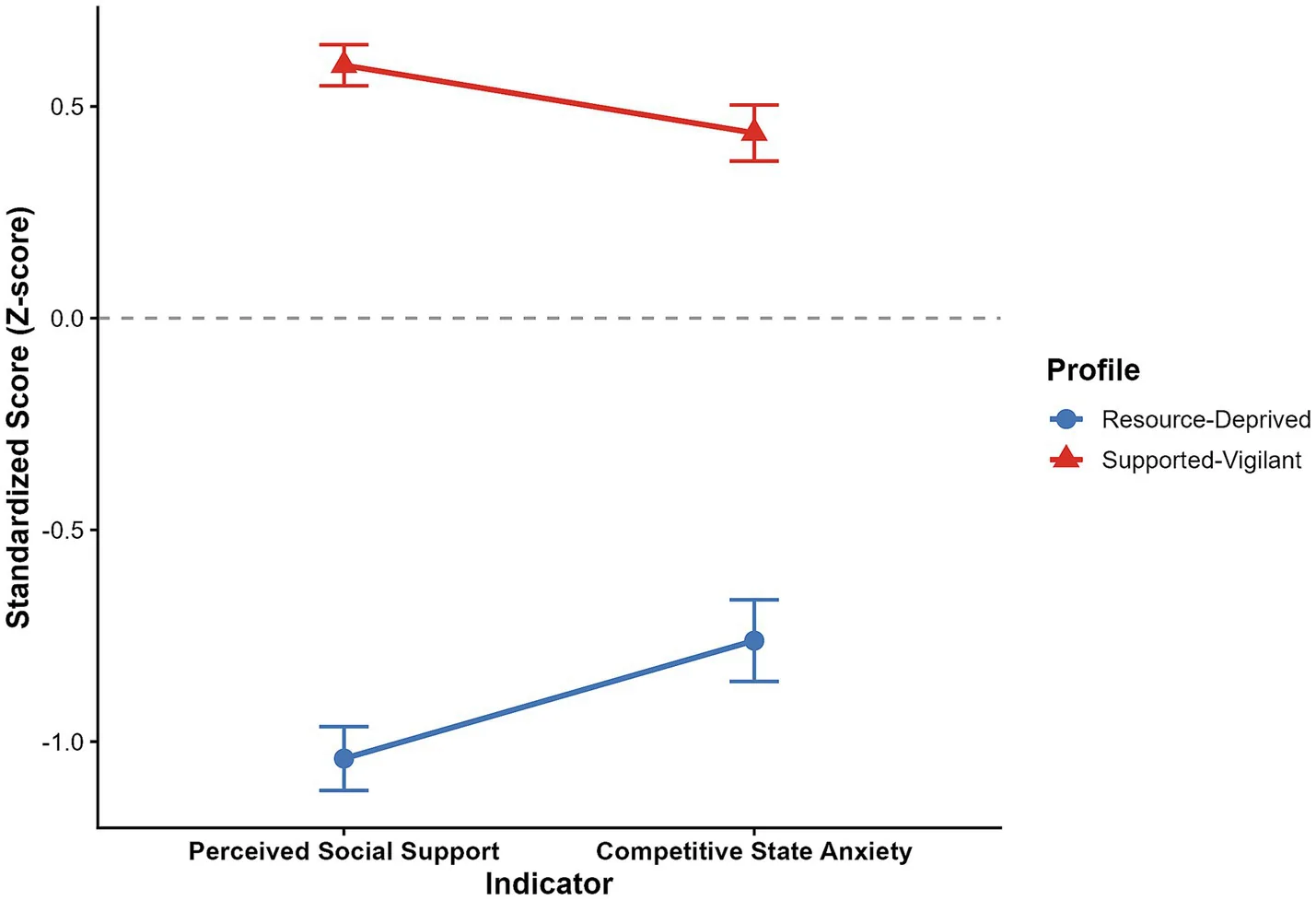
Latent profiles on standardized perceived social support and competitive state anxiety (*N = 2*22, entropy = 0.679; error bars represent ±1 SE).

The standardized mean profiles for the two-profile solution are presented in Table 4 and Figure 2. *Profile 1 — Resource-Deprived Profile (n = 81, 36.5%)*: Athletes in this profile scored below the full-sample mean on both perceived social support (*z* = −1.04) and competitive state anxiety (*z* = −0.76). Thus, these athletes reported comparatively limited perceived support together with below-average competitive state anxiety. *Profile 2 — Supported–Vigilant Profile (n = 141, 63.5%)*: Athletes in this profile scored above the full-sample mean on both perceived social support (*z* = 0.60) and competitive state anxiety (*z* = 0.44). Thus, this profile was characterized by a combination of greater perceived social support and above-average competitive state anxiety.

**Table 4:** Standardized mean profiles for the latent profiles.

Profile	Perceived social support (*z*)	Competitive state anxiety (*z*)	*n*	Proportion
1 Resource-Deprived	−1.040	−0.762	81	36.5%
2 Supported–Vigilant	0.598	0.437	141	63.5%

### 3.4 Profile differences in depression and mental toughness

**Figure 3:**
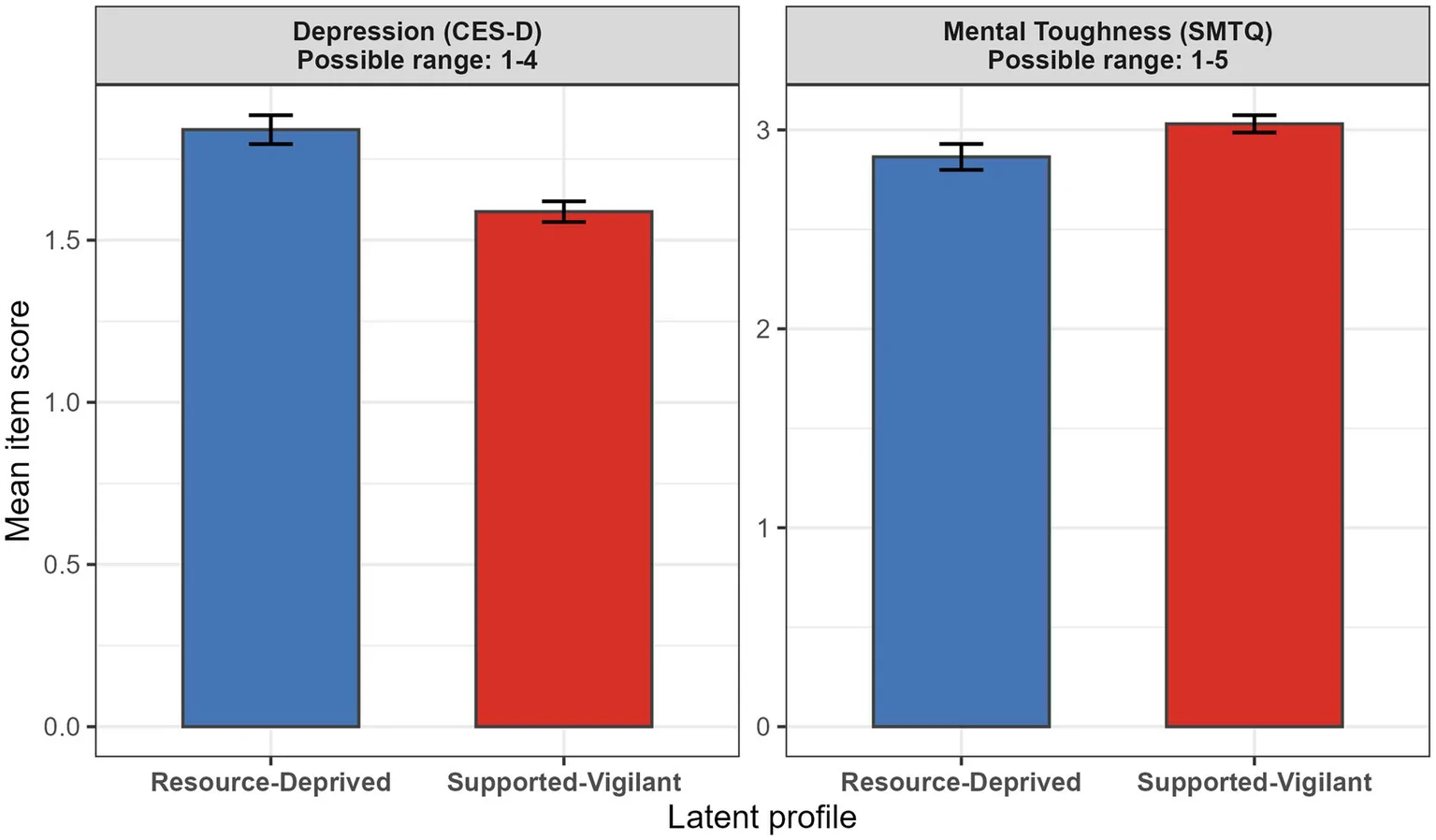
Mean item scores for depression and mental toughness by profile (error bars = ± 1 SE).

The results of the independent-samples *t*-tests are presented in Table 5 and Figure 3. The Supported–Vigilant profile, characterized jointly by higher perceived support and higher competitive state anxiety, reported significantly lower depression than the Resource-Deprived profile (1.588 vs. 1.841, t = 4.62, *p* < 0.001) and significantly higher mental toughness (3.030 vs. 2.864, t = −2.13, *p* = 0.035). These findings indicate that the higher-support/higher-anxiety configuration was associated with a more favorable pattern of external psychological correlates—lower depression and higher mental toughness.

**Table 5:** Differences between the two latent profiles in depression and mental toughness (independent-samples *t*-tests).

Outcome variable	Resource-deprived (*M*)	Supported-vigilant (*M*)	*t*	*p*
Depression (CES-D)	1.841	1.588	4.62	< 0.001
Mental toughness (SMTQ)	2.864	3.030	−2.13	0.035

### 3.5 Covariate-adjusted correlates of profile membership

**Figure 4:**
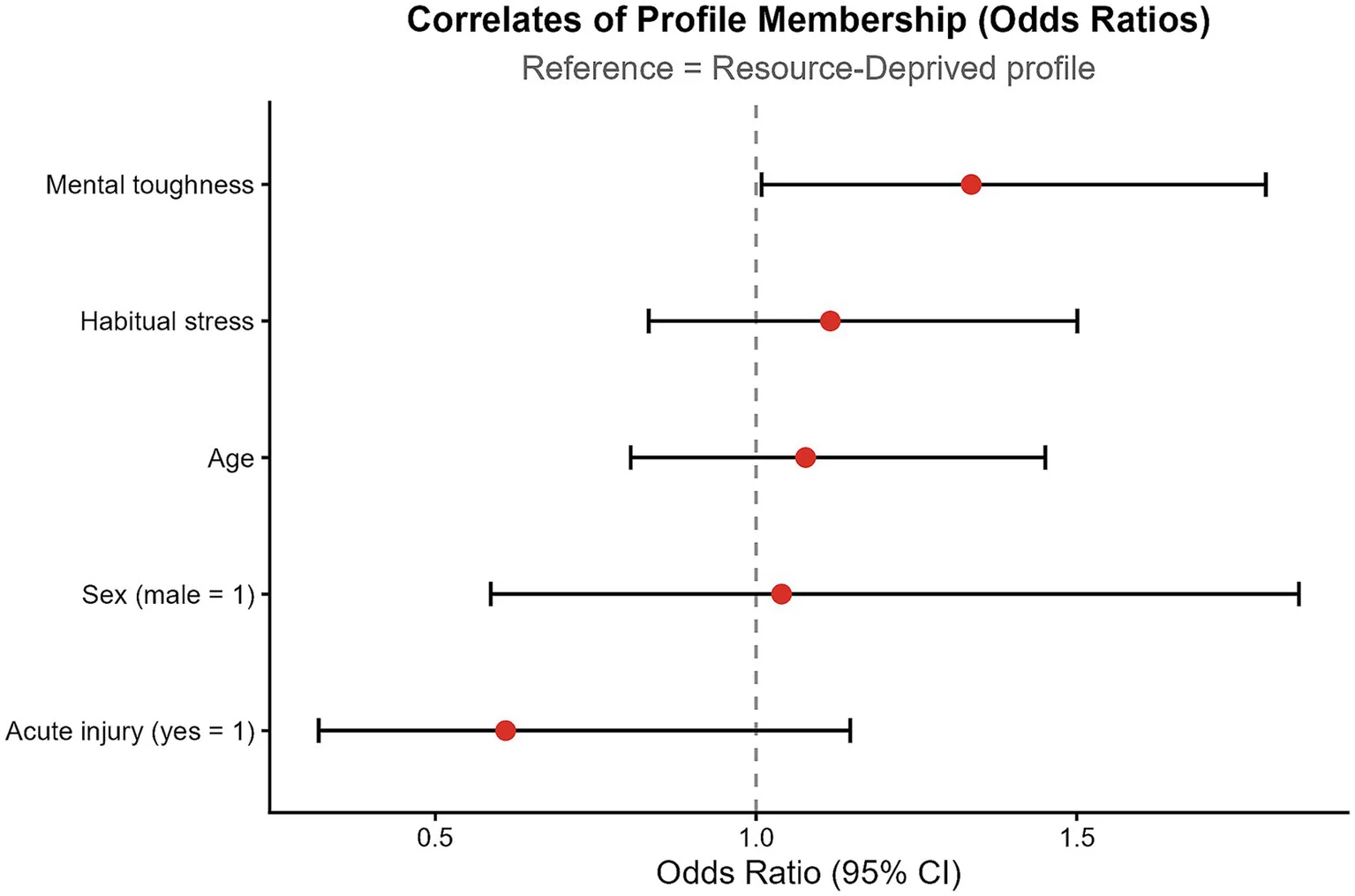
Covariate-adjusted correlates of profile membership (odds ratios and 95% confidence intervals; reference = Resource-Deprived).

The results of the binary logistic regression, with the Resource-Deprived profile (Profile 1) as the reference group, are presented in Table 6 and Figure 4. After adjustment for habitual stress, age, sex, and acute injury, mental toughness was significantly associated with higher odds of membership in the Supported–Vigilant profile (OR = 1.34, 95% CI [1.01, 1.80], *p* = 0.047). Each one-standard-deviation increase in mental toughness was associated with 34% higher odds of membership in the Supported–Vigilant profile. Habitual stress was not significantly associated with profile membership (OR = 1.12, 95% CI [0.83, 1.50], *p* = 0.464); nor were sex, age, or acute injury.

**Table 6:** Covariate-adjusted associations with profile membership (reference group = Resource-Deprived).

Variable	OR	95% CI lower	95% CI upper	*p*
(Intercept)	3.91	1.34	12.01	0.015
Mental toughness (*z*)	1.34	1.01	1.80	0.047
Habitual stress (*z*)	1.12	0.83	1.50	0.464
Age (*z*)	1.08	0.81	1.45	0.619
Sex (male = 1)	1.04	0.59	1.85	0.894
Acute injury (yes = 1)	0.61	0.32	1.15	0.129

### 3.6 Demographic distribution differences across profiles

Chi-square tests showed no significant distributional differences between the two profiles in sex (χ^2^ = 0.002, *p* = 0.961), only-child status (χ^2^ = 0.214, *p* = 0.644), monthly household income (χ^2^ = 0.560, *p* = 0.756), judo grade (χ^2^ = 5.141, *p* = 0.273), acute injury (χ^2^ = 2.143, *p* = 0.143), or chronic injury (χ^2^ = 1.058, *p* = 0.304). Thus, none of the measured background variables showed statistically significant distributional differences across the two profiles (see Table 7).

**Table 7:** Distributional differences across profiles on background variables (chi-square tests).

Background variable	χ^2^	df	*p*
Sex	0.002	1	0.961
Only-child status	0.214	1	0.644
Monthly household income	0.560	2	0.756
Judo grade	5.141	4	0.273
Acute injury	2.143	1	0.143
Chronic injury	1.058	1	0.304

## 4 Discussion

Using person-centered latent profile analysis, the present study identified two pre-competition psychological profiles among young Chinese judo athletes, jointly defined by perceived social support and competitive state anxiety, namely the Resource-Deprived profile and the Supported–Vigilant profile. The Supported–Vigilant profile, characterized by higher standardized levels of both perceived support and composite competitive state anxiety, showed lower depression and higher mental toughness than the Resource-Deprived profile. This pattern is inconsistent with the simple assumption that lower anxiety invariably coincides with better psychological adjustment and is compatible with—although it does not directly test—the directional perspective on competitive anxiety (15, 19).

The retained two-profile solution itself warrants consideration. Although the information criteria generally decreased as additional profiles were estimated, each of the three- to five-profile solutions contained a very small profile comprising only three athletes (1.4% of the sample), raising concerns about subgroup stability and substantive interpretability. The two-profile solution was therefore retained on the basis of class size, parsimony, and interpretability, rather than because it produced the lowest BIC. The two indicators were moderately positively correlated (*r* = 0.56), and the retained profiles primarily distinguished athletes with jointly lower versus jointly higher levels of perceived social support and composite competitive state anxiety. More differentiated configurations, such as high support combined with low anxiety or low support combined with high anxiety, were not reliably recovered in the present sample. A two-profile structure is not unprecedented in person-centered athlete research; for example, previous resilience-profile research also retained two athlete profiles (13), whereas research based on a broader set of emotional indicators has identified three or more profiles (21). These differences suggest that the number and granularity of profiles may depend partly on the breadth of the indicators and the characteristics and size of the sample. With *N* = 222, the present study may have had limited capacity to recover small or more differentiated subgroups reliably. Future studies using larger samples and additional indicators, such as anxiety direction, challenge/threat appraisal, and coping, are needed to determine whether more differentiated configurations can be replicated.

Traditional variable-centered research has tended to yield the linear conclusion that “stronger social support, lower anxiety, and better adaptation go together” (8, 30). However, the profile-based evidence in the present study reveals a more nuanced picture: among athletes characterized by relatively strong social support (the Supported–Vigilant profile), higher composite competitive state anxiety was not accompanied by higher depression or lower toughness but instead coexisted with lower depression and higher mental toughness. This pattern is theoretically compatible with the directional theory of anxiety, according to which whether elevated arousal impairs performance and well-being depends on whether the athlete interprets it as facilitative or debilitative (16, 31). One possible explanation is that athletes who perceive strong social support may be more inclined to appraise competition as a “challenge” rather than a “threat” (10, 31), in which case their higher pre-competition arousal might be framed as “combat readiness” rather than a “threat signal.” In combat sports, evidence has shown that self-confidence and the regulation of emotional arousal positively predict self-rated performance via challenge appraisal (19), providing a theoretical context for the present observation that higher composite competitive state anxiety coexisted with more favorable external correlates in the higher-support profile. In other words, one theoretical possibility is that social support may not simply “suppress” anxiety but rather change the psychological meaning of that anxiety. The present pattern is also broadly compatible with the “facilitative-arousal” profile in athlete emotion research: among profiles defined by pre-competition emotional intensity and direction, the facilitative-arousal profile, despite having a far-from-low arousal level, reports stronger challenge appraisals and greater perceived control (21). In the highly combative discipline of judo, the present study is therefore consistent with, but does not directly substantiate, the proposition that “high arousal is not necessarily maladaptive” through the configuration of social support and anxiety. Importantly, neither anxiety direction, challenge/threat appraisal, nor combat readiness was measured in the present study; these accounts should therefore be understood as theoretically informed possible explanations rather than conclusions derived directly from the data.

In contrast to the “high-anxiety, high-functioning” Supported–Vigilant profile, athletes in the Resource-Deprived profile were “doubly low” on both social support and anxiety, yet reported higher depression and lower mental toughness. Their lower composite competitive state anxiety therefore did not coincide with better psychological adjustment in this sample. One possible, but untested, interpretation is that this lower anxiety reflects reduced engagement or withdrawal against a backdrop of resource depletion. This possible interpretation is consistent with the “anhedonia/amotivation” presentation associated with adolescent depression (32) and accords with the theory that social support serves as a protective resource for toughness—when external support is scarce, individuals lack the psychological resources needed to transform stress into resilience (33). Alternative explanations, however, cannot be excluded: some athletes may have attached less subjective importance to the upcoming competition, held more modest competitive goals, been at an earlier stage of competitive development, or differed in response style. Because engagement, motivation, perceived competition importance, anhedonia, and these alternative explanations were not directly assessed, all such accounts remain speculative. The Resource-Deprived profile may therefore warrant particular attention in pre-competition psychological intervention, and its comparatively low anxiety may be overlooked in routine interventions that take “anxiety reduction” as their sole objective.

The logistic regression results showed that mental toughness was significantly associated with higher odds of membership in the Supported–Vigilant profile relative to the Resource-Deprived profile. This is consistent with findings from resilience-profile research, in which high-resilience athletes tend to report higher perceived social support, better health behaviors, and greater well-being (13). Mental toughness and perceived social support may be related in mutually reinforcing ways, but their temporal ordering cannot be determined from the present cross-sectional data (33). The association between habitual stress and Supported–Vigilant profile membership did not reach statistical significance (*p* = 0.464) and should therefore be interpreted cautiously. Prior variable-centered research has tested a mediational mechanism whereby perceived social support influences mental toughness via depression (34), characterizing a general pathway applicable to all athletes; the present study, by contrast, suggests heterogeneity in the joint distribution of these psychological variables: two empirically distinguishable configurations were observed, although the data do not establish that the profiles follow qualitatively distinct causal pathways.

This study carries the following implications. First, pre-competition psychological work should move beyond an exclusive “one-size-fits-all” focus on “reducing anxiety.” For Supported–Vigilant athletes, higher composite competitive state anxiety coexisted with lower depression and higher mental toughness; however, this does not establish that their anxiety was facilitative or that it reflected combat readiness. Practitioners should therefore consider anxiety appraisal, perceived support, confidence, and broader psychological adjustment before determining whether anxiety-reduction strategies are appropriate. Second, identifying and prioritizing intervention for Resource-Deprived athletes, who report comparatively low anxiety yet higher depression and lower mental toughness, may be particularly important; building social support from coaches, teammates, and family for these athletes may represent a potential intervention approach whose effectiveness should be evaluated. Third, future intervention studies could examine whether toughness training may serve as a potential pathway for helping athletes transition from the Resource-Deprived profile to the Supported–Vigilant profile.

This study has several limitations. First, the cross-sectional design cannot establish temporal or causal direction; therefore, the associations between profile membership and the external variables examined require confirmation in longitudinal or intervention studies. Second, all variables were self-reported and may be subject to biases such as social desirability. Third, the profiles were based on only two indicators—social support and anxiety; neither the facilitative–debilitative direction of anxiety nor challenge/threat appraisal or combat readiness was measured. Therefore, the interpretive accounts offered above remain tentative, and future work could incorporate the directional dimension of anxiety and dedicated appraisal measures to test the “framing” hypothesis proposed here more directly. Fourth, the entropy of the two-profile solution was 0.679, indicating moderate classification accuracy and some classification uncertainty; moreover, although models with additional profiles were estimated, these solutions produced very small profiles comprising only three athletes (1.4% of the sample). The sample size (*N* = 222) may therefore have limited the reliable detection of more finely differentiated subgroups, and the generalizability of the findings requires validation in larger and more diverse samples. Fifth, the auxiliary analyses were based on most-likely profile assignments and did not explicitly adjust for classification uncertainty; therefore, the resulting between-profile differences and adjusted associations should be interpreted cautiously.

## 5 Conclusion

The retained two-profile solution comprised a Resource-Deprived profile and a Supported–Vigilant profile, distinguished primarily by jointly lower versus jointly higher levels of perceived social support and composite competitive state anxiety. The Supported–Vigilant profile reported lower depression and higher mental toughness despite its higher composite competitive state anxiety; however, these findings do not establish that its anxiety was facilitative, adaptive, or buffered by social support. Conversely, the Resource-Deprived profile reported higher depression and lower mental toughness and may therefore warrant particular attention in pre-competition psychological assessment and support. Mental toughness was significantly associated with higher odds of membership in the Supported–Vigilant profile. These results support a person-centered perspective on athletes’ pre-competition psychology and point toward individualized assessment and support strategies that consider perceived social support, anxiety intensity, and broader psychological adjustment together.

## Data Availability

The raw data supporting the conclusions of this article will be made available by the authors, without undue reservation.

## References

[1] Ciaccioni S Perazzetti A Magnanini A Kozsla T Capranica L Doupona M. Intergenerational judo: synthesising evidence- and eminence-based knowledge on judo across ages. Sports (Basel). (2024) 12:177. doi: 10.3390/sports12070177, 39058068 PMC11281159

[2] Ciaccioni S Castro O Bahrami F Tomporowski P Capranica L Biddle S . Martial arts, combat sports, and mental health in adults: a systematic review. Psychol Sport Exerc. (2024) 70:102556. doi: 10.1016/j.psychsport.2023.102556 37949383

[3] Racine N McArthur BA Cooke JE Eirich R Zhu J Madigan S. Global prevalence of depressive and anxiety symptoms in children and adolescents during COVID-19: a meta-analysis. JAMA Pediatr. (2021) 175:1142–50. doi: 10.1001/jamapediatrics.2021.2482 34369987 PMC8353576

[4] Lee YH Chiu W Hwang J Noh S. Mobile-based mindfulness meditation intervention’s impact on mental health among young male judo athletes in South Korea: a quasi-experimental study. Sci Rep. (2024) 14:12691. doi: 10.1038/s41598-024-63637-0, 38830986 PMC11148055

[5] Artioli GG Gualano B Franchini E Scagliusi FB Takesian M Fuchs M . Prevalence, magnitude, and methods of rapid weight loss among judo competitors. Med Sci Sports Exerc. (2010) 42:436–42. doi: 10.1249/MSS.0b013e3181ba8055, 19952804

[6] Lakicevic N Roklicer R Bianco A Mani D Paoli A Trivic T . Effects of rapid weight loss on judo athletes: a systematic review. Nutrients. (2020) 12:1220. doi: 10.3390/nu12051220, 32357500 PMC7281976

[7] Miarka B Brito CJ Amtmann J Córdova C Dal Bello F Camey S. Suggestions for judo training with pacing strategy and decision making by judo championship phases. J Hum Kinet. (2018) 64:219–32. doi: 10.1515/hukin-2017-0196, 30429913 PMC6231334

[8] Luo J Du R Wang X Luo L. The relationship between social support and mental health in athletes: a systematic review and meta-analysis. Front Psychol. (2025) 16:1642886. doi: 10.3389/fpsyg.2025.1642886, 40969465 PMC12442422

[9] Fletcher D Sarkar M. A grounded theory of psychological resilience in Olympic champions. Psychol Sport Exerc. (2012) 13:669–78. doi: 10.1016/j.psychsport.2012.04.007

[10] Meijen C Turner M Jones MV Sheffield D McCarthy P. A theory of challenge and threat states in athletes: a revised conceptualization. Front Psychol. (2020) 11:126. doi: 10.3389/fpsyg.2020.00126, 32116930 PMC7016194

[11] Shi C Wang Y Chen J Wang Z Gao X Fan Y . Effects and mechanisms of social support on adolescent athletes’ engagement. Sci Rep. (2025) 15:7018. doi: 10.1038/s41598-025-92110-9 40016349 PMC11868561

[12] Clough PJ Earle K Sewell D. "Mental toughness: the concept and its measurement". In: Cockerill I, editor. Solutions in Sport Psychology. London: Thomson Learning (2002). p. 32–43.

[13] Chrétien A Hayotte M Vuillemin A d'Arripe-Longueville F. Resilience profiles of elite athletes and their associations with health-related behaviors, well-being, and performance: a latent profile analysis. Psychol Sport Exerc. (2024) 74:102689. doi: 10.1016/j.psychsport.2024.102689 38901549

[14] Wu Z Luo F Liu X Li J Zhou Y Luo J. Examining the effects of competitive state anxiety and goal orientation on sports performance of college track and field athletes. Front Psychol. (2025) 16:1607747. doi: 10.3389/fpsyg.2025.1607747, 40584061 PMC12203609

[15] Jones G Swain A Hardy L. Intensity and direction dimensions of competitive state anxiety and relationships with performance. J Sports Sci. (1993) 11:525–32. doi: 10.1080/02640419308730023, 8114178

[16] Jones G. More than just a game: research developments and issues in competitive anxiety in sport. Br J Psychol. (1995) 86:449–78. doi: 10.1111/j.2044-8295.1995.tb02565.x 8542197

[17] Jones G Hanton S Swain A. Intensity and interpretation of anxiety symptoms in elite and non-elite sports performers. Personal Individ Differ. (1994) 17:657–63. doi: 10.1016/0191-8869(94)90138-4

[18] Hanton S Connaughton D. Perceived control of anxiety and its relationship to self-confidence and performance. Res Q Exerc Sport. (2002) 73:87–97. doi: 10.1080/02701367.2002.10608995, 11926488

[19] Morrone M Ventura L Roggio I Di Blasio A Ruiz M Deriu F . Predicting performance of elite kickboxers using the multi-states theory framework. Eur J Sport Sci. (2024) 24:e12031. doi: 10.1002/ejsc.12031

[20] Spurk D Hirschi A Wang M Valero D Kauffeld S. Latent profile analysis: a review and "how to" guide of its application within vocational behavior research. J Vocat Behav. (2020) 120:103445. doi: 10.1016/j.jvb.2020.103445

[21] Nogueira JM Morais C Mansell P Gomes AR. Emotional profile of athletes before competition: contributions for perceived stress, cognitive appraisal and coping strategies. Front Sports Act Living. (2025) 7:1636826. doi: 10.3389/fspor.2025.1636826, 40809101 PMC12345605

[22] Zimet GD Dahlem NW Zimet SG Farley GK. The multidimensional scale of perceived social support. J Pers Assess. (1988) 52:30–41. doi: 10.1207/s15327752jpa5201_2 2280326

[23] Yang X Xue M Pauen S He H. Psychometric properties of the Chinese version of the multidimensional scale of perceived social support. Psychol Res Behav Manag. (2024) 17:2233–41. doi: 10.2147/PRBM.S463245 38835653 PMC11149633

[24] Martens R Burton D Vealey RS Bump LA Smith DE. "Development and validation of the competitive state anxiety Inventory-2". In: Martens R Vealey RS Burton D, editors. Competitive Anxiety in Sport. Champaign: Human Kinetics Books (1990). p. 117–90.

[25] Chen Z Peng L. Associations between parental expectations and competitive state anxiety in adolescent tennis players: mediation by basic psychological needs. Children (Basel). (2025) 12:1714. doi: 10.3390/children12121714, 41462854 PMC12732109

[26] Herge WM Landoll RR La Greca AM. "Center for Epidemiologic Studies Depression Scale (CES-D)". In: Gellman MD Turner JR, editors. Encyclopedia of Behavioral Medicine. New York: Springer (2013). p. 366–7.

[27] Jiang L Wang Y Zhang Y Li R Wu H Li C . The reliability and validity of the Center for Epidemiologic Studies Depression Scale (CES-D) for Chinese university students. Front Psych. (2019) 10:315. doi: 10.3389/fpsyt.2019.00315, 31178764 PMC6537885

[28] Sheard M Golby J van Wersch A. Progress toward construct validation of the sports mental toughness questionnaire (SMTQ). Eur J Psychol Assess. (2009) 25:186–93. doi: 10.1027/1015-5759.25.3.186

[29] Wang H Gao H Yang C Zhao L. The relationship between coaching leadership behaviors and athletes’ psychological fatigue: the mediating role of psychological resilience. Front Psychol. (2026) 17:1828916. doi: 10.3389/fpsyg.2026.1828916, 42453450 PMC13368335

[30] Yang L Wang N Li D Zhao X Wen M Zhang Y . Social support and anxiety: a moderated mediating model. Sci Rep. (2025) 15:29390. doi: 10.1038/s41598-025-14336-x, 40790318 PMC12339740

[31] Jones M Meijen C McCarthy PJ Sheffield D. A theory of challenge and threat states in athletes. Int Rev Sport Exerc Psychol. (2009) 2:161–80. doi: 10.1080/17509840902829331 PMC7016194 32116930

[32] Watson R Harvey K McCabe C Reynolds S. Understanding anhedonia: a qualitative study exploring loss of interest and pleasure in adolescent depression. Eur Child Adolesc Psychiatry. (2020) 29:489–99. doi: 10.1007/s00787-019-01364-y, 31270605 PMC7103575

[33] Sarkar M Fletcher D. Psychological resilience in sport performers: a review of stressors and protective factors. J Sports Sci. (2014) 32:1419–34. doi: 10.1080/02640414.2014.901551, 24716648

[34] Chen W Wang H Liu Z Yao J Chu D Zhu X . The relationship between perceived social support and psychological resilience in Chinese adolescent judo athletes: a cross-sectional study on the mediating role of depression and the moderating role of age. Front Psych. (2025) 16:1558351. doi: 10.3389/fpsyt.2025.1558351, 41000345 PMC12457420

